# Low-latency multi-threaded processing of neuronal signals for brain-computer interfaces

**DOI:** 10.3389/fneng.2014.00001

**Published:** 2014-01-28

**Authors:** Jörg Fischer, Tomislav Milekovic, Gerhard Schneider, Carsten Mehring

**Affiliations:** ^1^Institute for Biology I, University of FreiburgFreiburg, Germany; ^2^CorTec GmbHFreiburg, Germany; ^3^Bernstein Center Freiburg, University of FreiburgFreiburg, Germany; ^4^Department of Bioengineering and Department of Electrical and Electronic Engineering, Imperial College LondonLondon, UK; ^5^Faculty for Applied Sciences, University of FreiburgFreiburg, Germany

**Keywords:** brain-computer interface (BCI), latency, parallelization, software architecture, multi-threading

## Abstract

Brain-computer interfaces (BCIs) require demanding numerical computations to transfer brain signals into control signals driving an external actuator. Increasing the computational performance of the BCI algorithms carrying out these calculations enables faster reaction to user inputs and allows using more demanding decoding algorithms. Here we introduce a modular and extensible software architecture with a multi-threaded signal processing pipeline suitable for BCI applications. The computational load and latency (the time that the system needs to react to user input) are measured for different pipeline implementations in typical BCI applications with realistic parameter settings. We show that BCIs can benefit substantially from the proposed parallelization: firstly, by reducing the latency and secondly, by increasing the amount of recording channels and signal features that can be used for decoding beyond the amount which can be handled by a single thread. The proposed software architecture provides a simple, yet flexible solution for BCI applications.

## 1. Introduction

Brain-computer interfaces (Wolpaw et al., 2002; Nicolas-Alonso and Jaime, 2012; Shih et al., 2012) translate neural signals into commands driving an external actuator (e.g. computer, prosthetic arm). This way, BCIs can restore movement and communication abilities of paralyzed patients (Birbaumer et al., 1999; Wolpaw and McFarland, 2004; Hochberg et al., 2006; Kim et al., 2011).

Decoding neuronal activity typically involves multivariate signal processing (multiple channels and multiple signal features) and linear or non-linear methods for classification and regression. Therefore, the overall decoding process can be computationally demanding. On standard desktop computers or laptops, the amount of computations a processor core can execute per time is limited and may be insufficient for BCI signal processing. One can alleviate such limitations by distributing calculations among multiple cores. A higher amount of computational power available for decoding can be beneficial for several reasons: (1) Processing of neural activity measurements at a higher spatio-temporal resolution, i.e. more recording channels and higher sampling rates, can potentially yield more accurate decoding of the subject's intentions (Carmena et al., 2003; Nicolelis et al., 2003; Gunduz et al., 2009). Moreover, the decoding accuracy can be improved by using multiple signal features from a single neuronal signal simultaneously, e.g. by decoding from multiple frequency bands of the same local field potential or electroencephalogram (EEG) channel (Rickert et al., 2005; Woon and Cichocki, 2007; Ang et al., 2008). In both cases, the computational demand increases with the number of channels or the number of features per channel. (2) More complex classification or regression algorithms can improve decoding accuracy, e.g. by incorporating non-linearities (Gao et al., 2003; Shpigelman et al., 2009) or by adapting to non-stationary neuronal signals (Rotermund et al., 2006; Blumberg et al., 2007; Wu and Hatsopoulos, 2008; Shpigelman et al., 2009). Such algorithms typically require a higher amount of computational power. (3) The computational load increases with the number of degrees of freedom (DOF) of the external actuator. For example, in the often used linear filter (Carmena et al., 2003; Hochberg et al., 2006; Schalk et al., 2008; Collinger et al., 2012), computational demand of the filter grows linearly with the number of DOFs. (4) Higher computational power can increase the number of decoding steps per second. Hence, BCI users can experience a smoother control and might, therefore, feel more comfortable with the BCI. Furthermore, by reducing the time between two decoding steps, the BCI user will receive faster feedback on the movements of the actuator, which can improve the performance (Cunningham et al., 2011). In contrast, long delays and low update rates can substantially decrease the user's comfort and performance while using the interface.

Here, we present an efficient BCI software architecture designed for multi-core processing of neuronal signals. Using a technique termed “independent substream parallelization”, we can divide the processing of neuronal signals into independent processing steps. Each of these steps can then be run as an individual thread, thereby making adequate use of multi-threading, prevailing protocol for parallel computations supported on desktop and laptop computers. Furthermore, we demonstrate that the performance of multi-threaded processing of neuronal signals is highly dependent on the waiting strategy, i.e. the algorithm used to exchange data between threads. Our results show a trade-off between the speed the data is exchanged (latency) and the processor (CPU) usage: low latency comes at the cost of high CPU usage and vice versa.

## 2. Methods

### 2.1. Architecture

We implemented our software architecture under C++ (Stroustrup, 2000) using the object-oriented programming paradigm. Main design requirement for our software architecture was to be modular, as this provides the following benefits: (1) it can be extended more easily and quickly. (2) since classes interact only via defined interfaces, all children of the same interface type can be interchanged via configuration. (3) frequently used modules, e.g. Fourier transformation or band pass filters, can be reused. (4) it is easier to test complex software configurations by first testing modules independently. These properties are particularly desirable for a BCI research software where modifications are done continuously and requirements can change quickly.

The structure of our software architecture can best be described through its domain model which describes our domain of interest in terms of function and data (Fowler, 2002) and consists of three central types of classes: (1) Data classes: classes that represent data, e.g. neuronal activity measurements or measurements of limb movements. One can also think of them as containers that serve to move values between other classes. (2) Processing classes: classes that generate or manipulate data, e.g. devices used to perform measurements (amplifiers, movement trackers, etc.) and algorithms for processing neural signals (low-/band-/high-pass filtering, classification, regression etc.). All processing classes have “start” and “stop” methods used to start and stop the operation of the class, as well as a method for querying their unique name, which is used to identify each object (the instantiation of a class is called an object). (3) Connection classes: classes that move data objects between processing objects. These connections are realized using the observer pattern (Gamma et al., 1994), which enforces *flexible* connections between processing objects, allowing connections to be changed during runtime.

We define a mode as a set comprised of processing and connection objects. One can have multiple modes in one BCI application instance and every mode can be selected, started and stopped. A typical mode, used in the BCI application, would be “open-loop calibration”, where movement data and neuronal signals are recorded for subsequent calibration of decoding algorithms. Another typical BCI mode would be “brain-control”, where the previously calibrated decoding algorithm is used to translate the subject's neural activity into commands for the external actuator. For practical purposes, all modes are configured using a configuration file. Each mode is created by first creating all the processing objects, and then, all the connection objects which are used to connect the processing objects. Source and destination objects for every connection are identified by their unique names. Connections (connection objects) between processing objects can be modified at runtime without the need to recompile the application.

In our software architecture, each processing object is able to run in a separate thread. Therefore, data objects can potentially be shared between threads, which can, if not handled properly lead to undefined behavior. One could make the access to data objects thread-safe by using synchronization data structures. This adds further complexity to the testing of data objects, as one would have to verify that no synchronization-related errors like deadlocks (Roscoe et al., 1997) and racing conditions (Roscoe et al., 1997) occur. To resolve this issue, one could apply a “copy on write” convention, i.e. requiring all processing objects to first create a copy of a shared data object and then apply modifications only to the copy. Although this approach can be implemented, we refrained from it for reasons of stability: if only one processing objects omits the copy step, e.g. because of a yet undiscovered programming error, the behavior of all other processing objects using the same shared object is undefined. We therefore resolved this problem by not sharing data objects at all. Instead, every processing object owns its own copy of the data object that is being modified. As consequence, every data object is derived from an interface requiring its heirs to implement a method that returns an identical copy of them. This approach simplifies the design of data objects since they do not need to handle multi-threaded access and modifications. In addition, the steps of copying and thread-safe passing of data objects can be performed identically by all processing objects. Thus, these operations had to be implemented only once and could subsequently be reused.

### 2.2. Degrees of BCI parallelization

The process of neural decoding used in BCIs can be seen as a sequence of signal processing steps applied to a stream of neural data. The processing steps vary depending on the signal type (EEG, electrocorticogram, spiking activity of individual neurons, etc.) and the BCI application. We call a single processing step a “filter” and the whole sequence of processing steps a “filter pipeline”. A “filter” is, therefore, a transformation (linear or non-linear) of an n-dimensional input signal to an m-dimensional output signal. With this terminology, low-pass, band-pass, or high-pass filters are included in the definition of “filters”, as well as the short-time Fourier transform (Allen, 1977) or classification and regression methods, such as linear discriminant analysis (Hastie et al., 2011), support vector regression (Vapnik and Chervonenkis, 1974) and Kalman filter (Kalman, 1960; Haykin, 2001).

**Figure 1:**
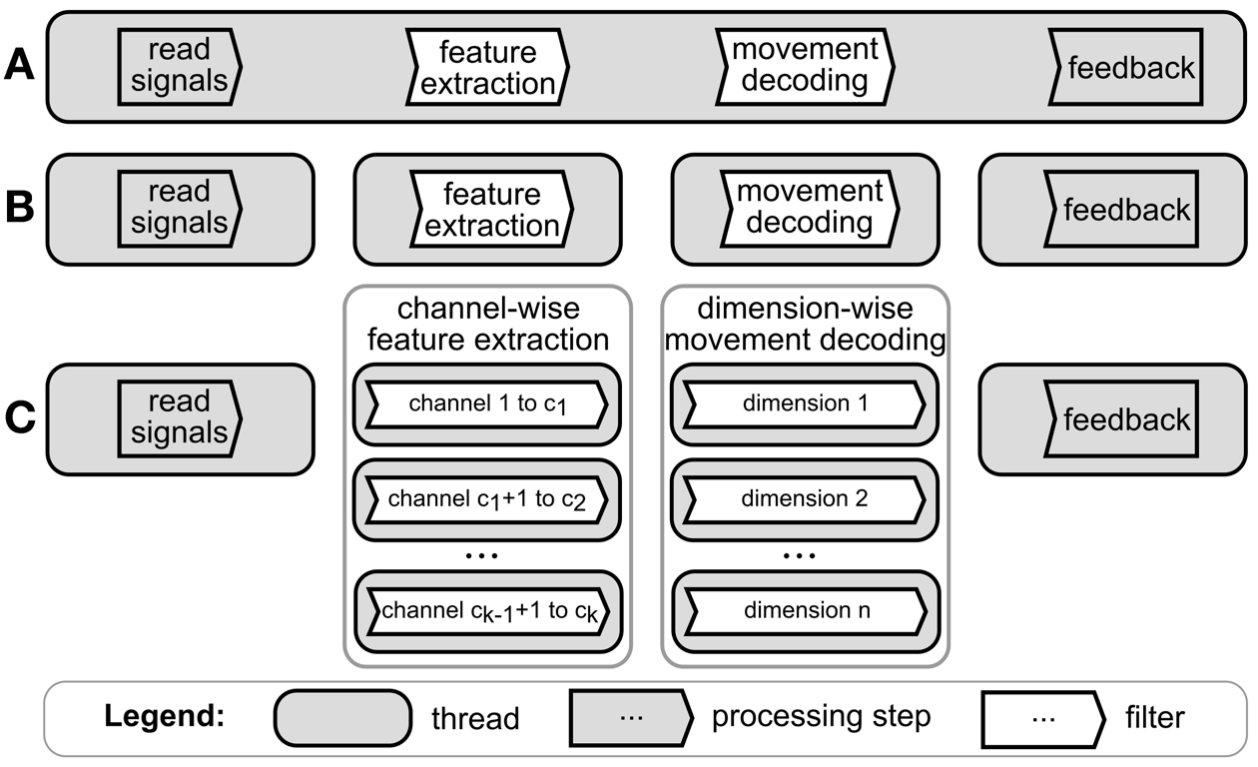
**Degrees of parallelization for a typical BCI software application**. Figure shows processing steps applied by a BCI application to a stream of neural data. **(A)** No parallelization, i.e. all processing steps are executed sequentially in one thread on a single CPU core. **(B)** Each processing step is executed in a separate thread. The computations of each thread can be handled by a different core. **(C)** Independent substream parallelization. Feature extraction and movement decoding steps are split into functionally independent computations, e.g. by computing the features for each channel in a different thread.

If all computations are executed in one thread, there is no parallelization, see Figure 1A. Such configuration can be used if no parallelization is needed, i.e. all computations are fast enough to be carried out by one thread before a new set of computations is requested. A first level of parallelization would divide the main processing steps, i.e. signal acquisition, feature extraction, decoding (classification or regression), and the generation of feedback, into separate threads (Figure 1B). This degree of parallelization can use as many cores as there are processing steps, since every thread can occupy only one separate core at any time. Existing facilities of the operating system, in our case the Windows scheduler, are used to distribute the workload of the threads among CPU cores. However, since each step depends on the result of the previous step, most computations are not carried out in parallel. In BCI software applications, many processing steps transform the signal on one channel independently from the signal on other channels (e.g. Fourier transformation or band-pass filters). This can be exploited for a higher degree of parallelization, where a processing step can be separated into functionally independent substreams, each processing a subset of channels. Each data substream can then be processed by a separate thread (Figure 1C, see Wilson and Williams (2009) for an example). We termed this level of parallelization as “independent substream parallelization”. The same principle can be extended to the processing of the decoding step. For example, if the BCI application is decoding the intended movement and each DOF of the movement can be computed independently from the other movement DOF (e.g. in a linear filter) then the decoding algorithms for each DOF can be run on a separate thread. There are ways to achieve even higher degrees of parallelization. In principle, all functionally independent parts of a processing step can be run on separate threads. However, such “fine grain” parallelization requires exploitation of the specific processing algorithm used. Therefore, there are no general ways to explore such ways of parallelization. Furthermore, current BCI systems process recordings from a large number of channels. Therefore, even by using “independent substream parallelization”, one can divide the processing into a number of substreams that far exceed the number of available CPUs used to run BCI software applications. We show that, when the number of threads is much greater than the number of available CPUs, one cannot expect additional gains in performance. For all these reasons, we did not consider parallelization on a degree finer than the “independent substream parallelization”.

### 2.3. Filter pipeline implementation

In this subsection we describe how we implemented the algorithmic concept of a filter in terms of software.

**Figure 2:**
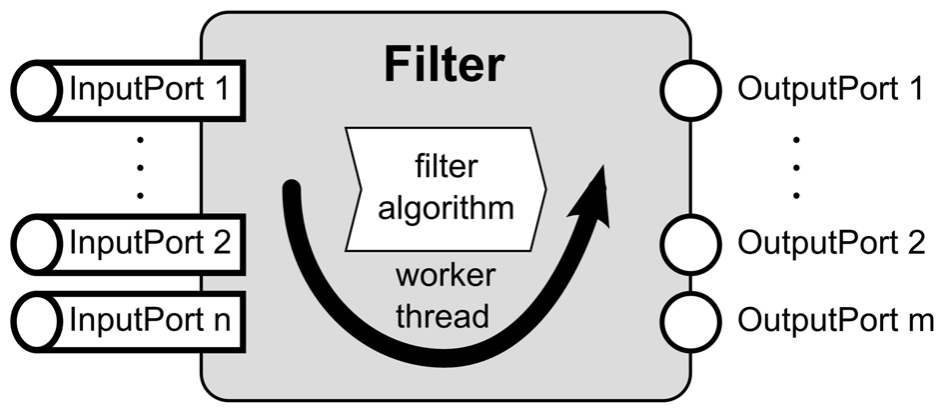
**Scheme of the filter implementation showing the main working steps of a filter**. After a filter is started, its worker thread collects the input data objects from all input ports. Then an exchangeable algorithm processes the data objects and its results are distributed to the output ports. This is repeated until the filter is stopped.

**Figure 3:**
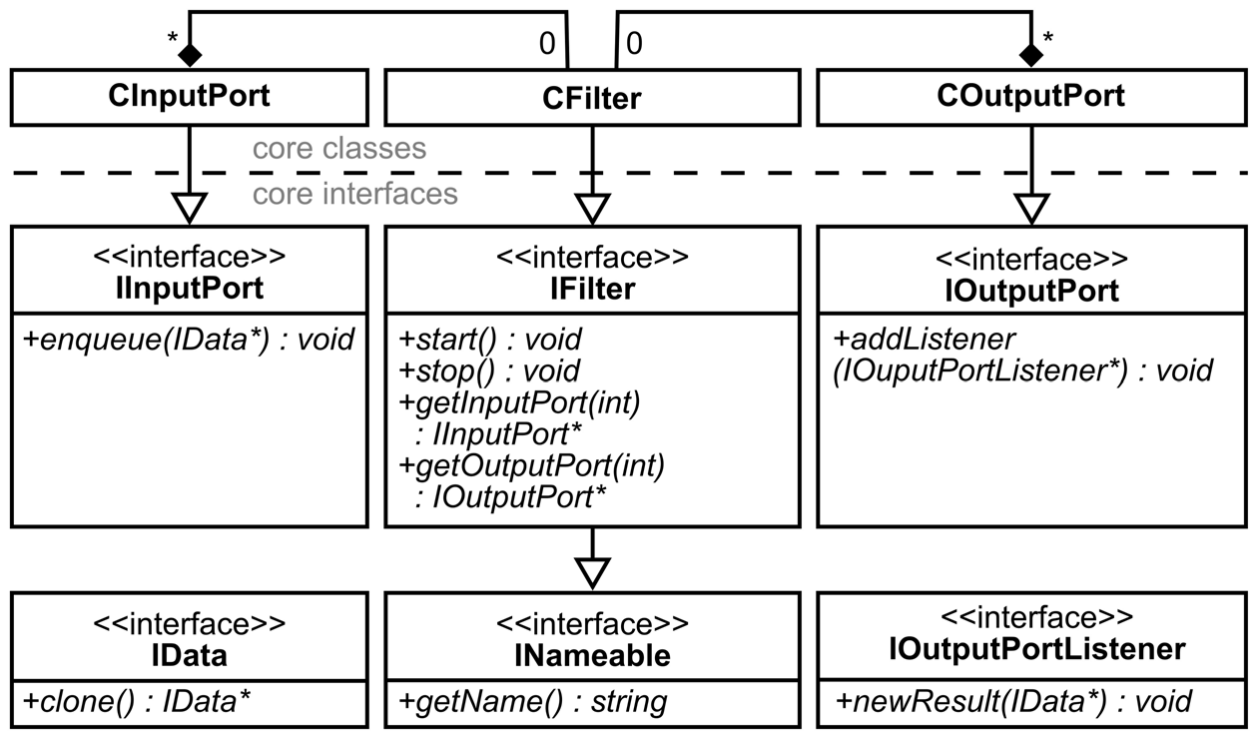
**Class diagram of the filter pipeline in unified markup language notation (Booch et al., 2005)**. Every IFilter implementation can have zero or more instances of CInputPort or COutputPort. IOutputPortListener is the interface from which connection classes inherit in order to receive data from an output port [cf. observer pattern in Gamma et al. (1994)].

Every filter has a set of input and output ports and an algorithm implementation which defines its function (Figure 2). Input ports are objects where the incoming data objects are handled in a thread-safe manner while preserving the order in which they arrived. In the output ports, data objects are sent to each connection object connected to the respective output port. A filter iterates three steps: (1) fetch one set of data objects from each input port, (2) process the data according to the algorithm implemented in the filter (filter algorithm in the remainder of the text), and (3) distribute resulting data objects through all output ports. To change the function of the filter, one only needs to exchange the filter algorithm. To facilitate the implementation of new filter algorithms, we implemented the IFilter^1^ interface with a class whose algorithm part is exchangeable through the IFilterAlgorithm interface. This design encapsulates all threading related functions, i.e. copying of data objects and passing them to other processing objects, in the surrounding filter class. From the developer's point of view, one can exclusively focus on the implementation of the actual filter algorithms, without having to care about the rest of the filter's functionality. Furthermore, our design allows developers to separately test their filter algorithms (IFilterAlgorithm). They can rely that the extensively tested remainder of the filter works properly and reliably. A more detailed class diagram of the key interfaces of the filter pipeline is shown in Figure 3.

There are different strategies on how a filter can check whether a complete set of data objects are available at each of the input ports (waiting strategies). We evaluated four waiting strategies. (1) Polling: repeatedly poll (i.e. query) the input ports for the new set of data objects, (2) Wait_0_: wait for the next time slice^2^ to check for the new set of data objects, (3) Wait_1_: wait for one period of the system event timer (16 ms in our system) to check for the new set of data objects and, (4) Wait_event_: wait until an event signal, sent by the input ports, is received and indicates that the new set of data objects is available. The source code of the filter pipeline with all waiting strategies is provided at the CorTec Homepage (2013) and can be used under the GNU General Public License version 3. We have successfully applied this software framework in a recent BCI study using the decoding of movement direction from the human electrocorticogram for closed-loop BCI control (Milekovic et al., 2012).

### 2.4. Simulation setup

We evaluated the performance of our BCI software architecture on a standard desktop personal computer (PC) with an Intel Core i7 970 computer with 3.2 gigahertz and 24 gigabyte memory running 64-bit Microsoft Windows 7 Enterprise operating system. The performance values will vary on different hardware and software configurations. However, we believe that the conclusions we draw from our quantitative results will be valid for a wide range of commonly used desktop and laptop computer systems. To measure the performance, we simulated a source of data that passed data objects to the filter pipeline. The artificial data source was a software simulation of a neurophysiological recording system, which, in real BCI applications, might record EEG, electrocorticogram signals, spike trains or other neuronal signals. In each simulation session, the data source provided 120 s of recordings from 256, 512, 768, or 1024 channels, sampled at a frequency of 256, 512, 768, or 1024 Hz. The filter pipeline consisted of one or more filters that processed the data (described in more detail below). The performance of the filter pipeline was quantified by three measures: (1) Median latency; For each waiting strategy *w* ∈ *W* := {Polling, Wait_0_, Wait_1_, Wait_event_} and number of threads, we measured the time required for the processing of a data object as the latency, i.e. the time between the injection of the data object into the filter pipeline and the reception of the resulting data object at the output of the filter pipeline. Since some of the algorithms considered in this study use a sliding window with a step size of *n* sample points, only every *n*-th input packet yields an output data packet and thus a latency value. Latency is denoted as *L*_*i,j*_, where *i* is the number of threads and *j* ∈ {1, …, *k*} is the *j*-th measurement of the latency. Due to computational processes initiated by the operating system, the processors used for simulations would occasionally be used for other computations. This would cause latencies much higher than expected for one or more consecutive measurement time points. To reduce the effect of such outliers, we reported median latency over all measurements. In addition, in each simulation, we removed the first 16 latency values (corresponds to 0.5 s of data) from each simulation in order exclude transient latency values at the start of the simulation. (2) Relative latency reduction (RLR); We define *N*_1_ := min_*w*∈*W*_ (median_*j*∈{1, …, *k*}_ (*L*_1,*j*_)) as the median latency of the waiting strategy with the minimum median latency when only one thread was used and use it as normalization factor to which we compare all other latencies to. For each latency *L*_*i,j*_, we further define RLR_*i,j*_ := *N*_1_/*L_i,j_*. By construction, RLR_*i,j*_ equals 1 if the latency measurement equals median latency of the fastest waiting strategy with one thread, is above 1 if *L*_*i,j*_ is smaller than *N*_1_ indicating an increase of performance relative to the fastest waiting strategy with one thread and is below 1 if *L*_*i,j*_ is greater than *N*_1_. To capture the general dependence of the latencies as a function of the number of threads, we define the median of relative latency reduction as MRLR_*i*_ := median_*j*∈{1, …, *k*}_ (RLR_*i,j*_). Thus, MRLR_*i*_ is larger than 1 if the computations are performed faster than with the fastest waiting strategy with one thread. (3) CPU load; measured once per second as the median percentage of the total possible load of one core (100%). We started CPU load measurement some time before the simulation started and thus initially measured CPU load from a period of inactivity. To exclude this period, we removed the first 2 CPU load measurements (corresponds to 2 s of data). Our test system contained six cores with Hyper Threading, which can allow two threads being processed in parallel on one core (Marr et al., 2002), so the maximum number of parallel threads, which can run at 100% CPU load, is 12. However, during our simulations, one thread simulated the data source and one thread was used to receive the results of the filter pipeline. In addition, some processing is required by the operating system. Therefore, the real number of threads that can be used exclusively by the simulated filter pipeline in parallel is lower than 12. We assumed that at least nine threads are available for full 100% CPU load, assuming that the operating system, the data source simulation and the acquisition of results from the simulation each occupy one thread at most.

A minimum requirement for a BCI application is that the filter can process the input data at least at the rate it is coming in. If processing is not fast enough, the data objects will accumulate at the input ports of a filter. Consequently, the latencies will add up, making the application unable to react to inputs. Additionally, the memory usage will grow until it reaches the limit of available memory, which will cause the application to terminate. We refer to this scenario as stall. During our simulations, a test run is considered to be stalled if one of the following conditions is true: (1) memory consumption exceeded a threshold of 750 megabytes (Mb) or (2) the overall calculation time exceeded the expected time by more than 1.2 s. The memory limit of 750 Mb is exceeded if more than 5 s of data is accumulated at the input port of one of the filters. We chose the memory limit big enough to reduce the number of falsely detected stalls as much as possible while giving the application sufficient remaining memory to shut down normally. If our system takes more than 1.2 s (1%) beyond the expected execution time of 120 s, it is fairly safe to conclude that the latency of the processing is too high. If the application would be allowed to continue to run, the data would accumulate and the program would eventually stall. While there is some necessary arbitrariness in the time limit of 1.2 s, it was chosen to reduce falsely detected stalls due to temporal jitter caused by other processes. Algorithms used in the BCI applications mostly fall into two groups: (1) feature extraction algorithms and (2) decoding (classification and regression) algorithms. In most BCI applications, these algorithms will take most of the computational time. Therefore, to evaluate the performance of our software architecture, we tested several feature extraction and decoding algorithms frequently used in BCI applications.

#### 2.4.1. Feature extraction algorithms

In our context, feature extraction algorithms are algorithms used to calculate features from neuronal signals. We used two frequently used feature extraction algorithms: short-time Fourier transform (Allen, 1977) and Savitzky–Golay filter (Savitzky and Golay, 1964; Milekovic et al., 2012) as an example of a linear filter for smoothing signals. Our implementation of the Savitzky–Golay filter is more demanding than our implementation of the short-time Fourier transform, since it uses one forward Fourier transform and one inverse Fourier transform in each calculation step.

#### 2.4.2. Classification and regression algorithms

BCI related decoding algorithms can be grouped into algorithms for inference of continuous variables (regression) and algorithms for inference of discrete variables (classification). For classification, we tested filter pipelines implementing linear discriminant analysis (LDA) (Hastie et al., 2011) and support vector machines (SVM) (Vapnik and Chervonenkis, 1974). For regression we tested the linear filter (LF), support vector regression (SVR) and the Kalman filter (KF) (Kalman, 1960; Haykin, 2001). We used libsvm library (Chang and Lin, 2011) to implement SVM and SVR, and the GNU Scientific Library (Gough, 2009) for linear algebra operations. For all tests of classification/regression algorithms, we kept the sampling frequency fixed at 1024 Hz and decoded one DOF, i.e. a one-dimensional movement. In the context of classification or regression algorithms, the computational complexity regarding the inputs is best measured by the number of features that have to be processed in each decoding step, rather than by the number of channels, as each channel might provide multiple features, e.g. data from multiple delays in respect to the current time or from multiple frequency bands. In addition to the computational complexity arising from the number of features, the computational complexity of the SVR and SVM algorithms depends on the number of support vectors. Therefore, we varied the number of support vectors for all tests involving the SVR and SVM algorithms. All feature extraction and decoding algorithms were executed 32 times per second (in steps of 31.25 ms).

#### 2.4.3. Complexity of classification and regression algorithms

For each algorithm, we derived parametric models of the latency as a function of feature dimension. For SVR and SVM, latency was also a function of the number of support vectors. These models were fitted to the measured latency values (see section 3.3) to estimate the parameter values. In each model description, *L* denotes the latency and *n_f_* the number of features and *n_s_* the number of support vectors. **LF/LDA:** One DOF decoding using LF models or binary classification using LDA models is equivalent to a scalar product between a feature vector and a vector of fixed coefficients (obtained by calibrating the model on the training data). Therefore, the computation time is linear in the number of features (Hastie et al., 2011). For LF and LDA decoders, we, therefore, modeled the latencies as *L* = *b* · *n_f_* + *a*. **KF:** In the limit of high number of features, the computation time of the Kalman filter algorithm is dominated by the inversion of a symmetric matrix. We used Cholesky factorization algorithm for matrix inversion (as the matrix to be inverted is symmetric) and the computational complexity of Cholesky factorization is a cubic function of a number of features (Santos and Chu, 2003). All other operations have linear or quadratic dependence on the number of features. Profiling our implementation of the Kalman filter revealed that, with *n_f_* = 128 and decoding one DOF, around 94% of the computation time in each iteration was used for the matrix inversion. Thus, all of our simulations were made in the regime of high number of features. Therefore, we neglected the contributions of all other Kalman filter calculations in our model of the Kalman filter latencies: *L* = *c* · *n*^3^_*f*_ + *a*. **SVR/SVM:** Computational costs of SVM/SVR scale as *O*(*n_f_* · *n_s_*) (Burges, 1998). Latencies were therefore modeled as *L* = *d* · *n_f_* · *n_s_* + *a*.

### 2.5. Statistical analysis

**Figure 4:**
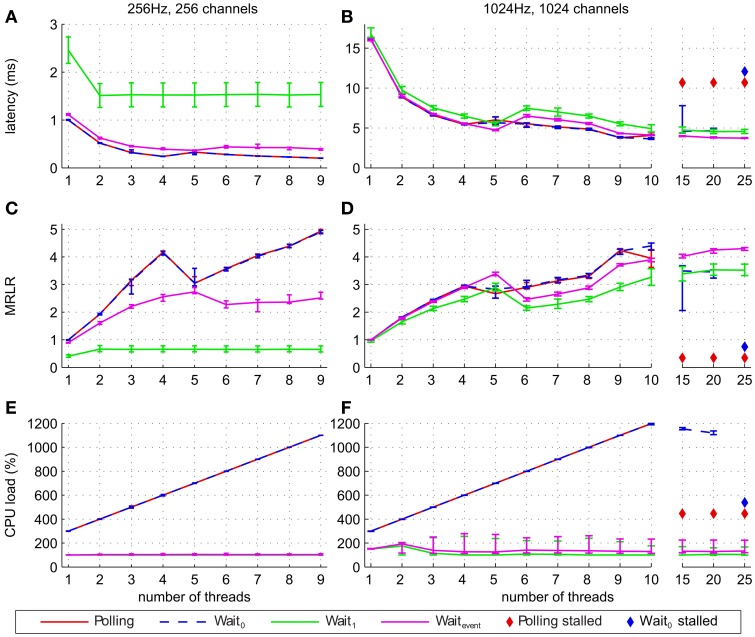
**Performance of the filter pipeline implementing the short-time Fourier transform algorithm for different waiting strategies and different number of threads**. Lines show median of latencies **(A,B)**, median of relative latency reduction **(C,D)**, and median CPU load **(E,F)**. The latencies, MRLR and CPU load of the waiting strategies Polling and Wait_0_ are very similar, so that their graphs mostly coincide. Error bars show the 25% and 75% percentiles. Panels on the left **(A,C,E)** show results for simulations with 256 Hz and 256 channels, while panels on the right **(B,D,F)** show results for simulations with 1024 Hz and 1024 channels. In addition, panels **(B,D,F)** (simulations with 1024 Hz and 1024 channels) show median latencies, MRLR and CPU load for 10, 15, 20, and 25 threads. For high number of threads a diamond symbol indicates that at least one out of ten simulations stalled when the corresponding waiting strategy was used.

We considered four different waiting strategies for the filter pipeline (see section 2.3) and measured their performance using three different measures: median latency, median of relative latency reduction and CPU load. Performance was examined as a function of the number of threads and across a range of sampling frequencies and numbers of channels (Figures S1–S3, Figure 4). For each combination of waiting strategy, number of threads, sampling frequency and number of channels, we ran ten simulations yielding 38,240 latency measurements and 1180 CPU load measurements in total. To assess the significance of the performance increase with increasing number of threads we compared the performance measures between one and nine threads by a Wilcoxon rank sum test (Hollander and Douglas, 1999; Gibbons and Chakraborti, 2011). The Wilcoxon rank sum test of MATLAB R2010a uses an approximation to compute *p*-values in the case of large samples. Given potential inaccuracies of this approximation we simply report “*p* < 0.0001” even if actual *p*-values were much smaller. We considered the null hypothesis as rejected if *p* < 0.05. This test was carried out for different sampling frequencies and number of channels (see section 3 and supplementary material).

## 3. Results

### 3.1. Performance of parallelization

Figure 4 shows latencies, MRLR and CPU load at the extreme points of our investigated parameter range (256 Hz/256 channels and 1024 Hz/1024 channels) for different number of threads. For all waiting strategies, except for Wait_1_, the latency generally decreased and the MRLR generally increased with more threads. Data was processed significantly faster when nine instead of one thread were used (*p* < 0.0001, Figure 4 and Figures S1, S2). Among all four waiting strategies, Wait_1_ yielded the poorest overall performance gain when more than one thread was used. This was particularly pronounced for low sampling frequencies and low channel numbers (see Figures S1, S2). The inferior performance of Wait_1_ is due to querying for data at fixed intervals of 16 ms, which can be highly suboptimal if the data arrived at the input port just after the last query, thereby causing a higher latency increase compared to the other waiting strategies. Wait_event_ performs better for higher sampling frequencies and number of channels (Figures 4B,D and Figures S1, S2). Compared to Wait_0_ and Polling, Wait_event_ performed worse for 256 Hz and 256 channels (*p* < 0.0001), whereas for 1024 Hz and 1024 channels Wait_event_ performed similar for up to four threads (median latency of Wait_event_ differs at most 0.15 ms, *p* < 0.0001), better for five threads (*p* < 0.0001) and slightly worse for up to nine threads (*p* < 0.0001, Figures 4A–D). Our latency and MRLR measurements show a drop in performance for five threads for Polling and Wait_0_ and for six threads for Wait_event_ and Wait_1_ waiting strategies. We suspect that the combination of the system hardware and the scheduler of Windows 7 caused the system to perform slower for some particular number of threads (e.g. five threads for Polling and Wait_0_ waiting strategies and six threads for Wait_1_ and Wait_event_ strategies), depending on the number of available cores. To test this hypothesis we reran our simulations on a four-core machine and indeed found the same drop in performance for three and four threads, respectively. A further test using the same hardware running Windows XP instead of Windows 7 revealed that the performance drop disappeared under Windows XP. Taken together, these additional tests support our initial presumption that the performance drop of Polling and Wait_event_ is caused by Windows 7 when the number of used threads is equal or one less than the number of available cores. We further explored the possible improvements in performance for the number of filter threads of up to 25, well above the total number of threads that can be used in parallel exclusively for filter pipeline simulation on our six-core Hyper Threading system. In this regime, several threads will compete for the use of different cores. We ran these simulations for a sampling frequency of 1024 Hz and 1024 channels to get a lower bound estimate of MRLR and an upper bound estimate of latency and CPU load (Figures 4B,D,F). While the performance of Wait_event_ and Wait_1_ further increased, MRLR of Wait_0_ decreased when number of threads was greater or equal to 15 and simulations stalled when 25 threads were used. Polling performance decreased for more than 10 threads and simulations stalled for 15, 20, and 25 threads (Figures 4B,D,F). CPU load of Polling and Wait_0_ strategies increased linearly with the number of threads until for 10 and more threads, where the maximum CPU load was observed. For the Wait_event_ and Wait_1_ strategies, CPU load remained at its low level of below 200%, even for up to 25 threads (Figures 4E,F). Therefore, for 10 and more threads, performance of the system may be influenced by the insufficient processing resources.

### 3.2. Stalls of the filter pipeline

**Figure 5:**
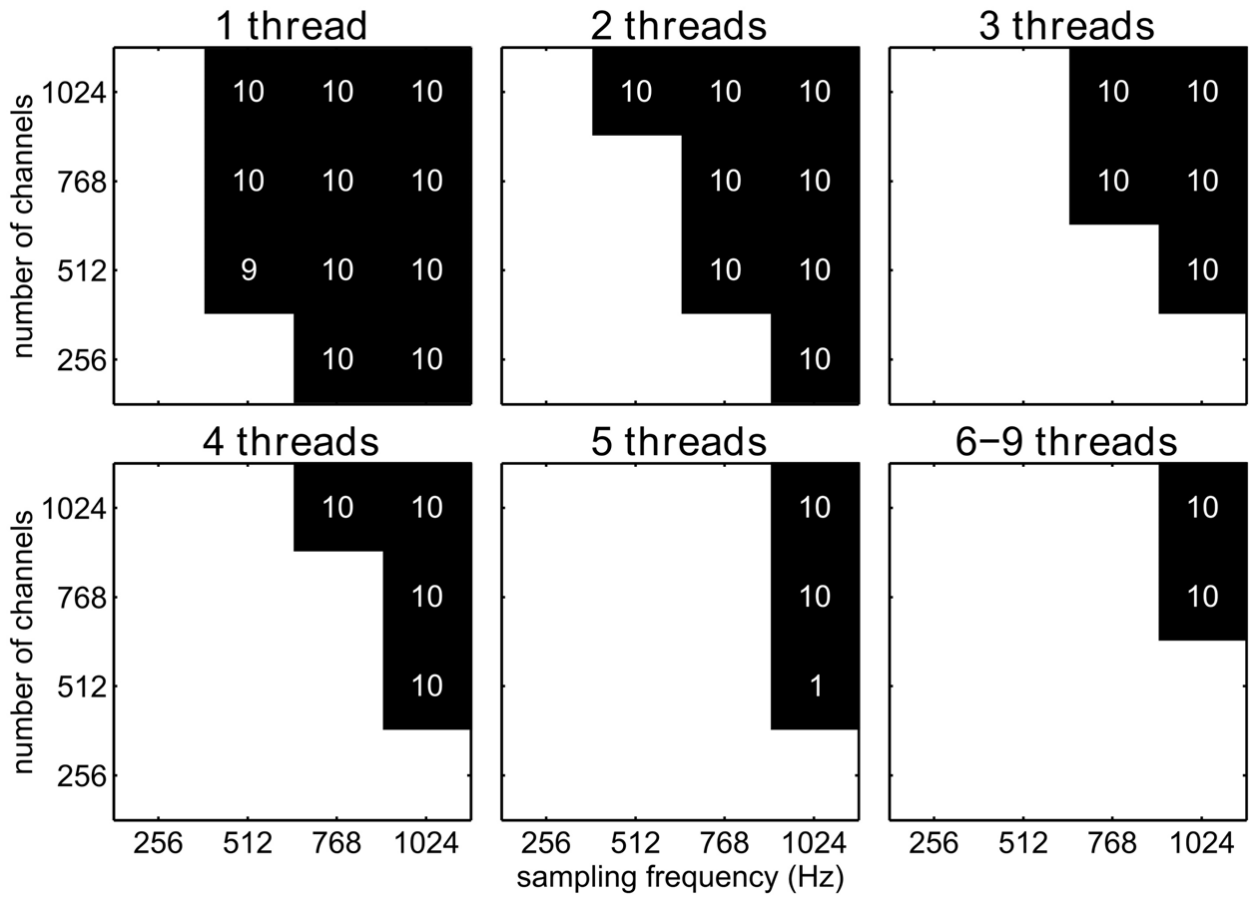
**Stalls for a filter pipeline implementing the Savitzky–Golay algorithm and using the Wait_event_ waiting strategy, shown for different channel number/sampling frequency combinations and different numbers of threads (1–9)**. A black square indicates combinations for which at least one out of ten simulations stalled, while white square stands for no stalls. Numbers in black squares indicate how many stalls out of ten simulations occurred.

If the processing of one decoding step exceeds the amount of time available during two consecutive decoding steps, data will accumulate at the input ports of the filter pipeline and, therefore, the system will eventually get increasingly delayed and run out of memory. We will call this incident a stall (see section 2.4). We investigated under which circumstances a stall can occur, when the application requires the complete translation of the neuronal signals to control signals (i.e. feature extraction and decoding) to be performed every 32 ms. Such a decoding rate is typical for continuous control BCI applications and lets the BCI user experience a smooth and virtually instantaneous control. For each number of channels and sampling frequency, 10 simulations were run. We assumed that a certain combination of number of channels and sampling frequency can be handled if no stalls were detected in all ten repetitions. For all tested sampling frequencies and for all tested numbers of channels, Fourier transform algorithm could be handled by only one thread. Due to the computationally more demanding Savitzky–Golay filter algorithm, implemented as a forward and inverse FFT, the Savitzky–Golay filter resulted in stalls for various parameter settings (Figure 5). Stalls already occurred for moderate sampling frequencies and for realistic number of channels if only one thread was used. However, by increasing the number of threads, independent substream parallelization allowed us to handle an increasing number of channels and higher sampling rates without stalls.

### 3.3. Performance of classification/regression algorithms

We measured latencies for filter pipelines that implemented either 1 DOF regression (LF, KF and SVR) or binary classification (LDA and SVM) algorithms (see section 2.4.2 for details). Measurements were made for different number of features, while the sampling frequency was fixed at 1024 Hz. The Wait_event_ filter implementation was used for all of these simulations, since it provides a good compromise between performance (latency reduction) and efficient CPU usage (cf. section 3.1).

**Figure 6:**
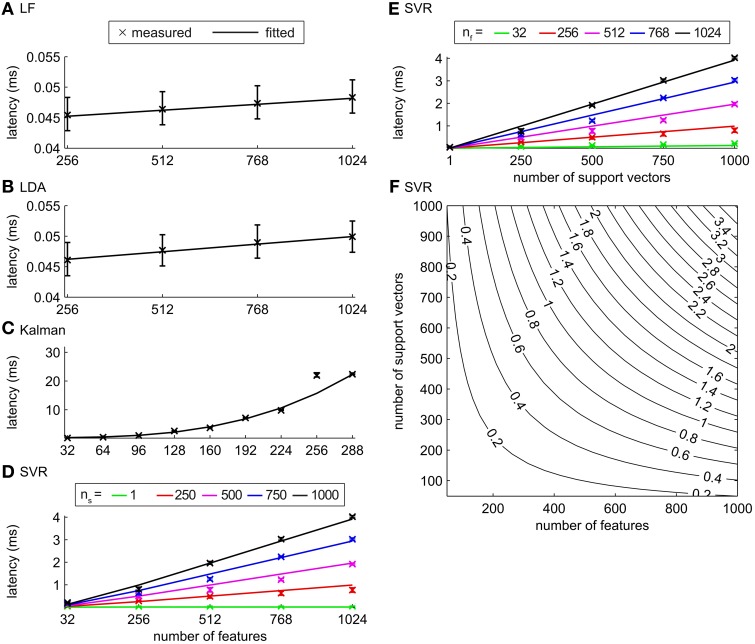
**Latencies for classification/regression algorithms. (A)** LF, **(B)** LDA, **(C)** KF, and **(D–F)** SVR. Crosses depict median latencies and error bars show 25% and 75% percentiles. Solid lines show fits of algorithm specific models to the measured values. **(D)** Latencies for the SVR algorithm as a function of the number of features for *n_s_* = 1, 250, 500, 750, and 1000. **(E)** Latencies for the SVR algorithm as a function of the number of support vectors for *n_f_* = 32, 256, 512, 768, and 1024. **(F)** Latencies given by the model of the SVR algorithm as a function of *n_f_* and *n_s_*.

The latencies of the LF and the LDA algorithms stayed below 0.1 ms for up to 1024 features, increasing slowly with the number of features (Figures 6A,B) with a linear model fitting the latencies well. The computation time of LF and LDA decoders was negligible compared to the short-time Fourier transform feature extraction (which required a computation time of more than 15 ms with one thread, 1024 Hz sampling frequency and 1024 channels) when our test system is used. The measured latencies of SVR increased linearly with the number of features and with the number of support vectors (Figures 6D,E). The measured latencies of SVM were almost identical to the latencies of SVR (Figure S4). The latency values for SVM and SVR (Figures 6D–F) were below 4 ms for all tested values of the number of features (up to 1024) and support vectors (up to 1000), far below the time needed for feature extraction. In summary, the parallelization of LF, LDA, SVM and SVR is, at least with the parameters and hardware considered here, not required. In contrast, the computation times of the KF algorithm reached values similar to the feature extraction for about 200 features (Figure 6C). For about 200 features and more the latency of the KF algorithm is in the range of the total time that is available for the decoding in a continuous BCI applications. In such cases, parallelization of the KF might thus be desirable. Due to the nature of the algorithm, KF cannot be parallelized using the independent substream parallelization suggested here. However, more complex parallelization schemes of the underlying computations could be employed (e.g. see Santos and Chu, 2003).

To estimate the latencies of the classification/regression algorithms for parameter values (e.g. number of features or support vectors) beyond the investigated values, we analyzed the computations that underlie each algorithm and derived for each algorithm a model that relates the latencies to the number of features and support vectors (see section 2.4.3 for details). The free model parameters were fitted to the measured latencies (Table 1, Figure 6, Figure S4). Using these models, we predicted the maximum number of features that can be handled within a given maximally allowed time by a tested decoding algorithm running in a single thread. Assuming that the maximal latency of the overall filter pipeline (feature extraction and decoding) should not exceed 32 ms for a smooth control, we allowed 10 ms (about one third) for the decoding algorithm, and allotted the remaining 22 ms for the feature extraction, other computations and as a safeguard against stalls. In these conditions, we can use up to 5.7 · 10^6^, 4.5 · 10^6^, and 286 features for LF, LDA, and KF, respectively and 57,625 and 57,958 features for SVR and SVM, if 100 support vectors are used for decoding. This further corroborates our finding that, in most currently realistic scenarios, all decoding algorithms except the Kalman Filter do not require parallelization. If the Kalman filter is used with hundreds of features, parallelization of the KF algorithm will increasingly become necessary to ensure smooth and instantaneous BCI control.

**Table 1:** **Fitted parameter values of the models describing the latencies of different classification/regression algorithms together with their lower and upper 5% confidence interval (CI) bounds**.

**Decoder**	**Coefficient**	**Lower CI**	**Value**	**Upper CI**
LF	a (μs)	44.22	**44.26**	44.30
	b (μs/feature)	0.00376	**0.00382**	0.00388
LDA	a (μs)	44.92	**44.96**	45.01
	b (μs/feature)	0.00478	**0.00484**	0.00490
KF	a (μs)	236.82	**238.74**	240.66
	c (μs/feature 3)	0.0009230	**0.0009231**	0.0009233
SVR	a (μs)	12.03	**12.36**	12.69
	d (μs/(feature · vector))	0.0038148	**0.0038156**	0.0038165
SVM	a (μs)	12.66	**12.98**	13.31
	d (μs/(feature · vector))	0.0037928	**0.0037936**	0.0037945

## 4. Discussion

In this article, we proposed a software architecture for BCI applications and evaluated its performance. The modular design of the proposed architecture makes it easy to parallelize typical processing steps of BCI applications by making use of multi-core computer processors and multi-processor configurations, which are nowadays available on standard desktop and laptop computers. The proposed domain model makes the integration of any type of processing algorithm simple. Algorithms are housed in a filter implementation, general and reusable modules of our architecture that enable immediate use of the parallelized data processing. We showed that, by using the proposed software architecture, it is simple to parallelize many time-consuming parts of the neuronal data processing in typical BCI applications. Our results demonstrate that using multiple threads in BCI signal processing leads to a substantial reduction of computing time required for one decoding step, further corroborating the findings of Wilson and Williams (2009). Wilson et al. evaluated the effects of multi-threading on computation time for two feature extraction algorithms, in one case using independent substream parallelization. We examined different implementations of multi-threading for BCI systems, i.e. different waiting strategies, and showed that the choice of the waiting strategy is crucial for CPU load and latency reduction. This reduction in latency determines how fast a BCI reacts to the user's input, which can directly affect the BCI performance: lower latencies can lead to improved performance, while higher latencies can lead to deterioration of performance (Cunningham et al., 2011). In addition, BCI user may himself experience a control latency, which might reduce his motivation and convenience. In contrast, experience of a quickly reactive, smooth control may motivate the user. Moreover, by reducing the latency, the remaining time allotted for one processing step can be used to process recordings from additional channels, more signal features, signals recorded at higher sampling frequencies and to decode using computationally more complex algorithms. All of these possibilities can lead to an improvement in performance (Bansal et al., 2012; Milekovic et al., 2013) and, therefore, might substantially increase the user's convenience with the BCI system. Our findings also show that, for realistic number of channels and a realistic sampling frequency, the BCI signal processing task can already be too demanding for a standard desktop computer if no parallelization is used. Hence, without parallelization the delay of the BCI feedback would need to be increased, resulting in a less smooth and delayed control.

By using the proposed software architecture, one can easily split the time consuming parts of the processing into separate threads: This is particularly straightforward for feature extraction algorithms, which often can be applied independently to each channel (e.g. low-/band-/high-pass filter, Fourier transform). In this case, each channel or different groups of channels could be contained in separate filters and run in separate threads. Our results show that among all waiting strategies Polling and Wait_0_ provide overall the best scaling of latency reduction with increasing number of threads. However, these two waiting strategies result in the highest CPU load, leaving less computational resources for additional applications to run in parallel. Moreover, the number of threads has to be chosen carefully for these strategies as the BCI application can stall if the number of threads exceeds the number of available cores. In contrast, the waiting strategy Wait_event_ is not stalling and performance is still increasing even if the number of threads is higher than the number of cores. Therefore, fine tuning of the number of threads to the hardware specifications of the computing system is not necessary for Wait_event_ (at least up to twice the number of threads than the number of cores). In addition, Wait_event_ achieves the highest latency reduction among all waiting strategies. While the scaling of the latency with numbers of threads is slightly less good compared to Polling and Wait_0_ this maximal latency reduction can be obtained by using more threads than cores. As a further advantage of Wait_event_, the CPU load remains low even if high numbers of threads were used. The Wait_event_ waiting strategy might therefore be the preferred choice for many applications as it combines low latencies, with low CPU usage and robustness against stalls.

In our study we improved BCI performance by applying different waiting strategies to multi-threaded neuronal signal processing and decoding. The proposed software architecture is also useful for improving BCI performance as follows. (1) For multi-DOF regression multiple LFs and SVRs can be used. This increases the computation time approximately by a factor of the number of DOF. However, as the computations of these separate LFs and SVRs are independent for each dimension, they can, therefore, be parallelized in a straightforward way by using the proposed independent substream parallelization with each thread computing the LF/SVR for one dimension. Similarly, multi-class classification with *C* classes using SVM can be handled by *C* independent one-vs.-rest SVMs or by *C* · (*C* − 1)/2 pairwise SVMs which are also independent. Multi-class LDA requires the computation of *C* − 1 decision functions, all of which can be calculated independently. Therefore, LF and SVM algorithms can also be efficiently parallelized, using our software architecture. (2) A boosting approach (Hastie et al., 2011), where many, eventually weak, decoders are combined into one stronger decoder, promises to be suitable for BCI scenarios with noisy signals. As the individual decoders process the signals independently, the boosting approach can also directly be parallelized with our software architecture. (3) Another scenario might be an adaptive decoder (e.g. Shpigelman et al., 2009) where time-consuming adaptation operations are run in one or more background threads in addition to the thread running the decoder. (4) Neuronal signal decoding can also be temporally independent, i.e. separate and independent computations have to applied to the signals at different time points and these computations could be carried out by separate threads, see Wilson and Williams (2009).

It is possible to further parallelize the BCI application beyond the parallelization approaches presented here. For example, the processing on the level of the algorithm itself could be parallelized, e.g. by using OpenMP (2013). Such approaches are more complex than the solutions proposed here and require custom-made solutions for each filter. Further optimization could also be achieved by integrating computations on graphics processing units into our software architecture, which provide fast and optimized algorithms for matrix and vector operations (Wilson and Williams, 2009). Some algorithms, for example the Kalman filter, cannot directly benefit from independent substream parallelization as their most time-consuming computations are not substream independent. For these algorithms, custom solutions have to be implemented like using parallelized code for the required matrix/vector operations, e.g. Cholesky factorization as described in Santos and Chu (2003).

In summary, the proposed software architecture substantially increases the computational power available for BCI signal processing while reducing latency. The architecture runs on standard desktop PCs and laptops and makes use of their multi-core/multi-processor hardware. The effects of waiting strategies on latency and CPU load were evaluated. The proposed software architecture's modular design enables BCI researchers to quickly modify, extend and reuse existing algorithms, as well as to implement new algorithms for neuronal signal processing. The algorithms immediately benefit from parallelization without requiring the programmer to possess any knowledge about multi-threaded programming.

### Conflict of interest statement

Jörg Fischer is employed by CorTec GmbH and owns shares of this company. The other authors declare that the research was conducted in the absence of any commercial or financial relationships that could be construed as a potential conflict of interest.

## Footnotes

^1^In our class naming convention, interfaces start with an “I” and normal classes with “C”.

^2^A time slice is a certain amount of execution time a thread gets allocated from a scheduling algorithm. When this time is used up, the thread has to stop execution until it gets a new time slice allocated.

## Supplementary material

The following Figures S1–S3 depict median latencies, MRLR and CPU load as a function of the number of threads for different sampling frequencies (256–1024 Hz) and different number of channels (256–1024). All graphs show the results for the short-time Fourier transform algorithm. Independent of sampling rate and channel count, performance increased with increasing number of threads. Additionally, for all investigated sampling frequencies and number of channels the waiting strategy Wait_event_ yielded the best compromise between low-latency and low CPU usage. Hence, these figures demonstrate that the results presented in section 3.1 are valid for a wide range of different sampling frequencies and number of channels. Figure S4 demonstrates that the computing times of support vector machines (SVM; for classification) are very similar to the computing times of support vector regression (SVR; for regression) shown in Figures 6D–F.

Figure S1 **Latency of the filter pipeline implementing short-time Fourier transform algorithm for different waiting strategies and numbers of threads**. Each subplot shows median of latencies (lines) with 25% and 75% percentiles (error bars) for one combination of sampling frequency and number of channels.

Figure S2 **MRLR for the filter pipeline implementing the short-time Fourier transform algorithm for different waiting strategies and numbers of threads**. Each subplot shows MRLR (lines) with 25% and 75% percentiles (error bars) for one combination of sampling frequency and number of channels.

Figure S3 **CPU load of our test computer running data source simulator, module that receives the processed data and the filter pipeline implementing the short-time Fourier transform algorithm for different waiting strategies and number of threads**. Each subplot shows median of CPU load (lines) with 25% and 75% percentiles (error bars) for one combination of sampling frequency and number of channels.

Figure S4 **Latencies for the filter pipeline implementing the SVM algorithm**. Markers show median of measured latencies with error bars showing the 25% and 75% percentiles. Solid lines show latencies predicted by algorithm specific models. Models in **(A)** used the SVM algorithm with 1, 250, 500, 750, and 1000 support vectors. Models in **(B)** used the SVM algorithm with 32, 256, 512, 768, and 1024 features as input. **(C)** Model prediction of SVM latencies in milliseconds (numbers on the solid lines) as a function of number of features and number of support vectors. Model assumes a linear increase of latency with the product of *n_s_* and *n_f_*.
