# Supplementary figures and images for: Low-latency multi-threaded processing of neuronal signals for brain-computer interfaces
Source: Front Neuroeng. 2014 Jan 28;7:1. doi: 10.3389/fneng.2014.00001 (PMC3904078; doi:10.3389/fneng.2014.00001)

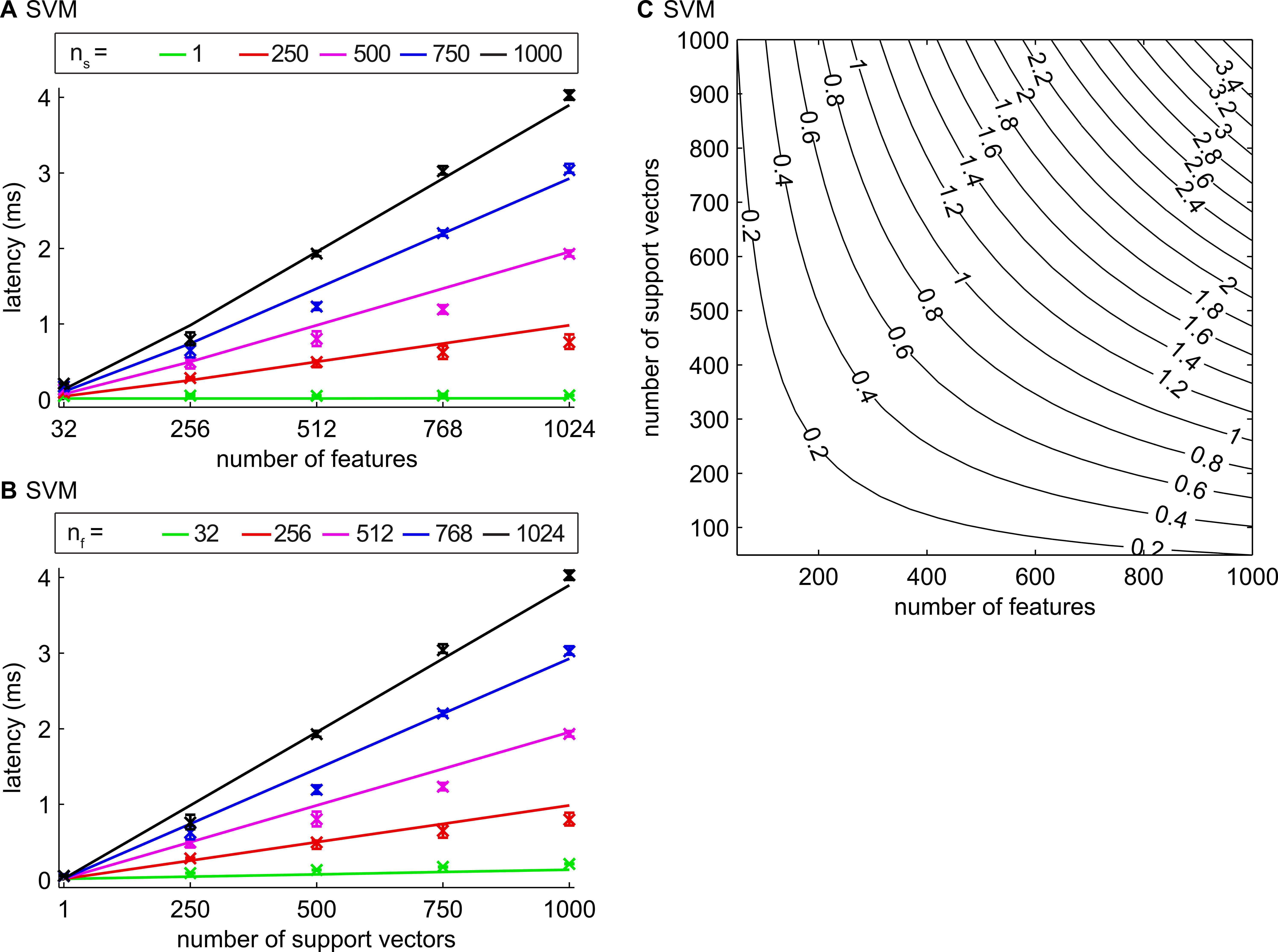

Supplement: Figure S1 — Latency of the filter pipeline implementing short-time Fourier transform algorithm for different waiting strategies and numbers of threads. Each subplot shows median of latencies (lines) with 25% and 75% percentiles (error bars) for one combination of sampling frequency and number of channels. [file Presentation1.ZIP › 65757_Fischer_Suppl Figure_4.TIF]

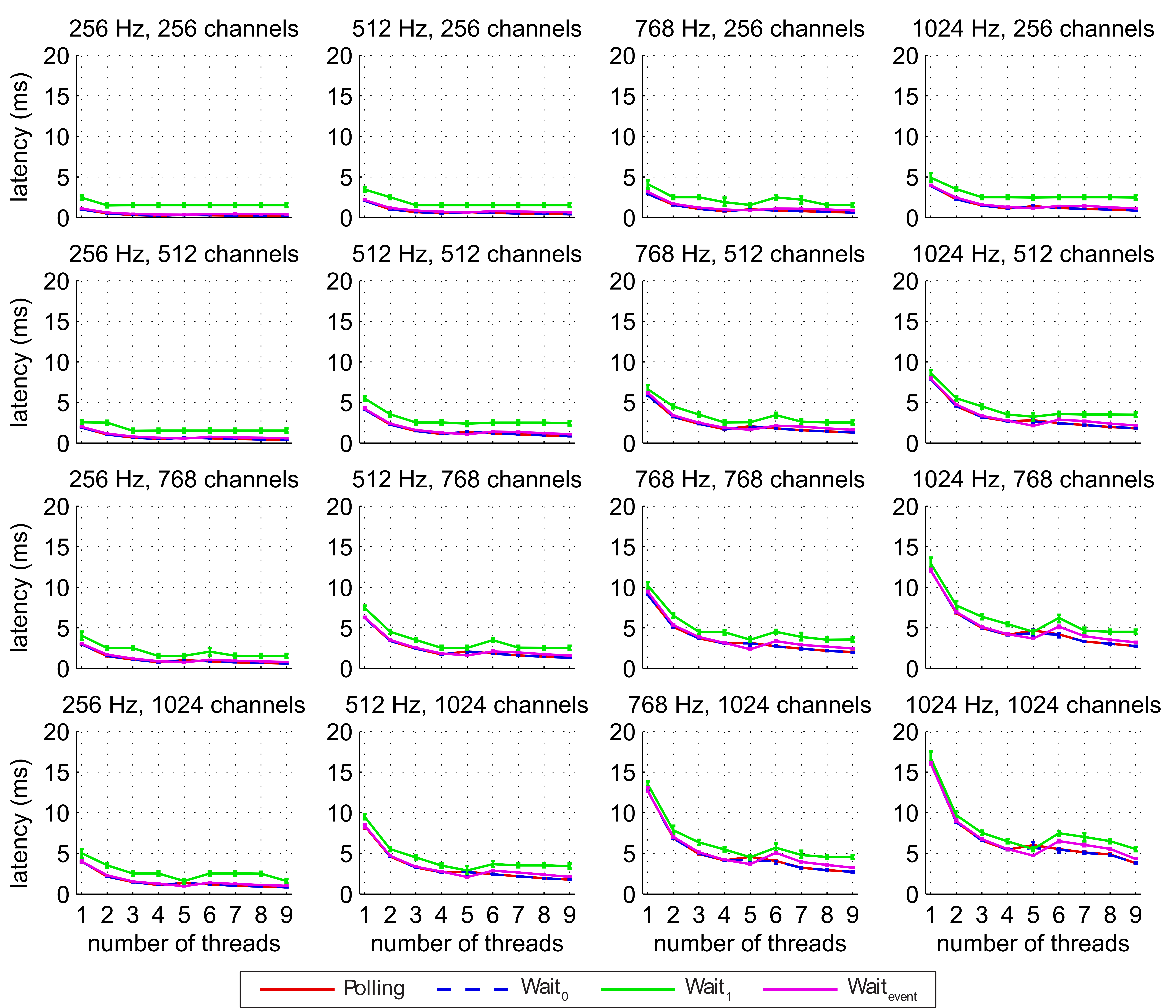

Supplement: Figure S1 — Latency of the filter pipeline implementing short-time Fourier transform algorithm for different waiting strategies and numbers of threads. Each subplot shows median of latencies (lines) with 25% and 75% percentiles (error bars) for one combination of sampling frequency and number of channels. [file Presentation1.ZIP › 65757_Fischer_Suppl Figure_1.TIF]

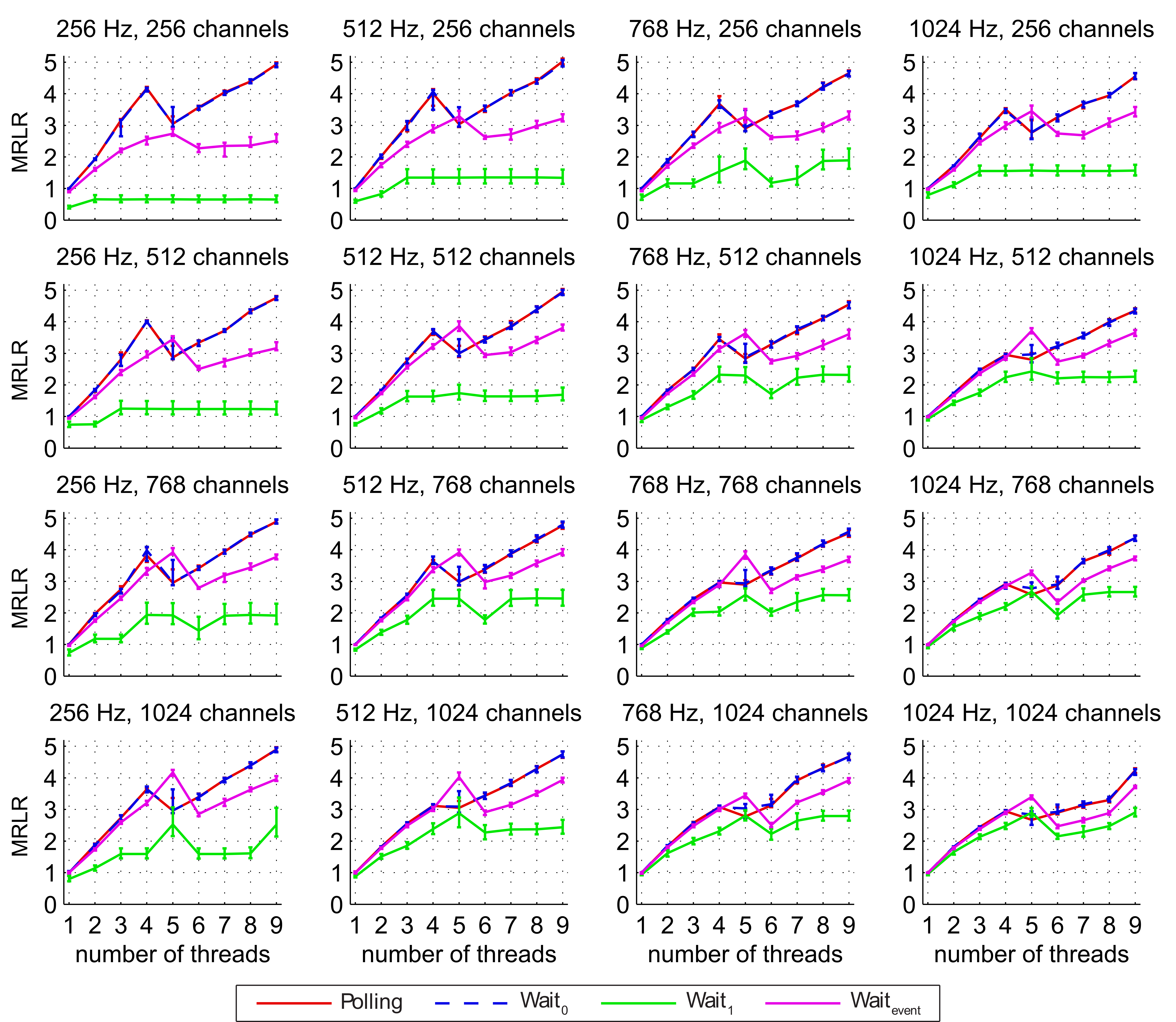

Supplement: Figure S1 — Latency of the filter pipeline implementing short-time Fourier transform algorithm for different waiting strategies and numbers of threads. Each subplot shows median of latencies (lines) with 25% and 75% percentiles (error bars) for one combination of sampling frequency and number of channels. [file Presentation1.ZIP › 65757_Fischer_Suppl Figure_2.TIF]

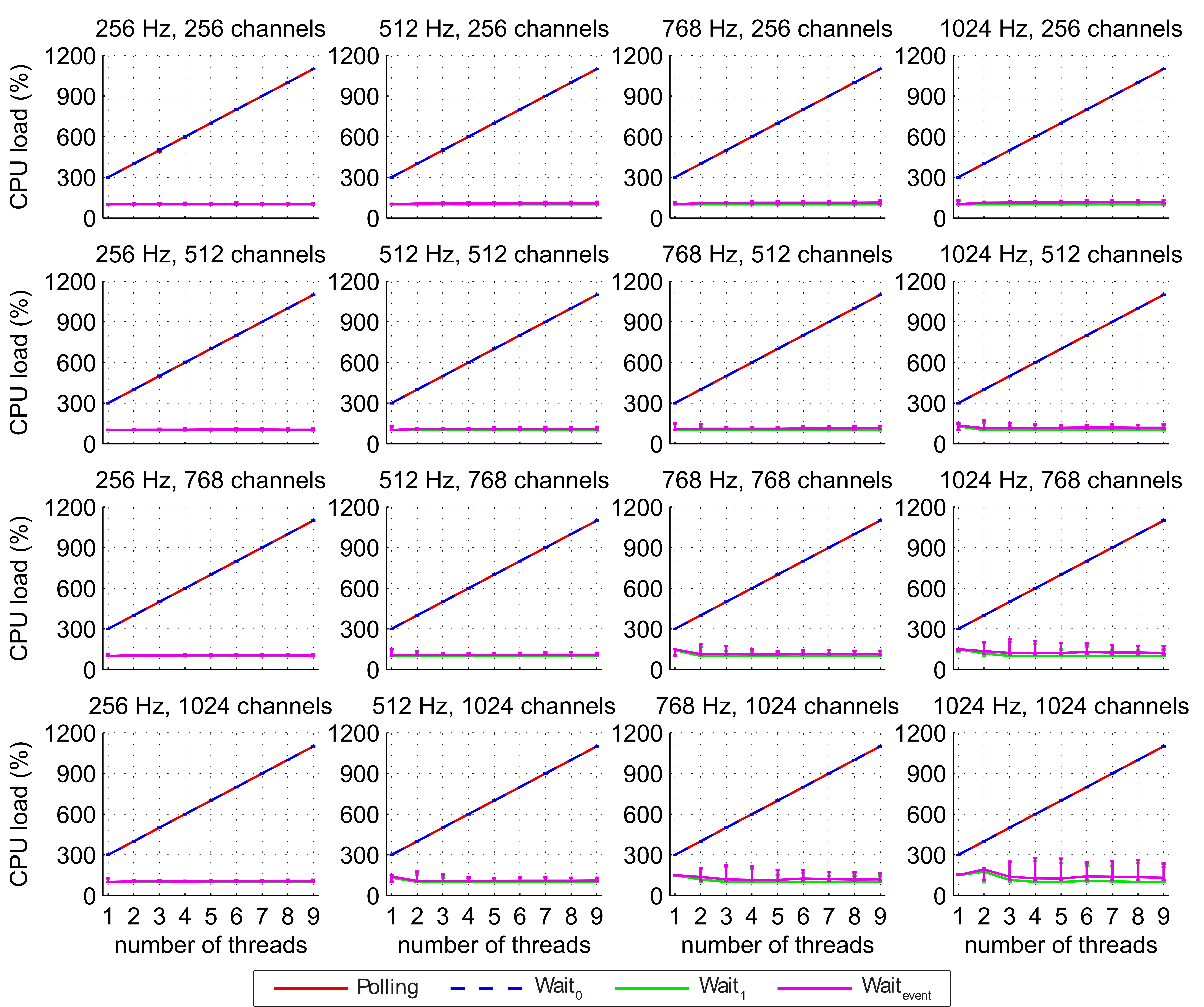

Supplement: Figure S1 — Latency of the filter pipeline implementing short-time Fourier transform algorithm for different waiting strategies and numbers of threads. Each subplot shows median of latencies (lines) with 25% and 75% percentiles (error bars) for one combination of sampling frequency and number of channels. [file Presentation1.ZIP › 65757_Fischer_Suppl Figure_3.TIF]
